# Protocol to produce and apply barcoded rabies virus for single-neuron input mapping in mice

**DOI:** 10.1016/j.xpro.2026.104537

**Published:** 2026-04-30

**Authors:** Kang Tan, Ya-qian Wang, Liu Fan, Hua-tai Xu

**Affiliations:** 1 Lingang Laboratory, Shanghai Center for Brain Science and Brain-Inspired Intelligence Technology, 555 Qiangye Road, Shanghai 201210, China

**Keywords:** Bioinformatics, Cell Biology, Cell culture, Neuroscience, Sequence analysis, Sequencing, Single Cell

## Abstract

Barcoded rabies viral tracing enables high-throughput mapping of brain-wide inputs to individual neurons while allowing integration with transcriptomic profiling. Here, we present a protocol to produce and apply barcoded rabies virus in mice for single-neuron input mapping. We describe steps to generate barcode plasmid libraries, produce barcoded rabies virus, and isolate target brain regions. We then detail procedures for preparing single-cell suspensions or bulk RNA, building barcode amplicon libraries from input regions, and performing initial analysis of the sequencing data.

For complete details on the use and execution of this protocol, please refer to Tan et al.^1^^,^^2^

## Highlights

• Instructions for preparing high-diversity barcoded rabies virus
• Guidance on preparing single-cell suspension from a small brain tissue sample
• Procedures for barcode amplicon library preparation
• Methods for aligning specific barcode patterns from sequencing data

## Abstract (editor highlights)

Publisher’s note: Undertaking any experimental protocol requires adherence to local institutional guidelines for laboratory safety and ethics.

## Graphical abstract

**Figure:**
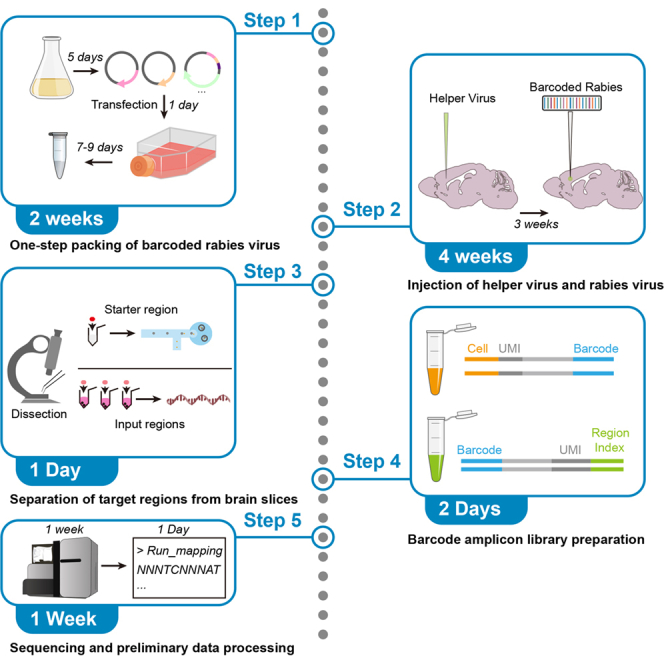

## Before you begin

This protocol outlines a complete workflow for the one-step production of high-diversity barcoded EnvA-pseudotyped rabies viruses, specifically SAD-B19ΔG and CVS-N2cΔG, followed by their application in the Barcoded Rabies Viral Tracing (BRT) method. Successful implementation relies on specific biological systems for both virus production and in vivo tracing. For virus generation, this protocol utilizes BHK-EnVA packaging cell lines expressing EnVA to ensure efficient amplification of the viral library.^3^^,^^4^ For the in vivo application, the protocol employs AAV helper viruses encoding the avian receptor (TVA) and rabies N2c-glycoprotein (N2cG) to genetically define starter populations within the mouse brain. While this protocol specifically describes mapping connectivity of mPFC neurons, the BRT method described here is adaptable to other brain regions and specific cell types where starter cells can be genetically targeted.

### Innovation

This protocol advances barcoded rabies viral tracing by providing a complete workflow that simplifies virus generation while preserving barcode diversity, a major limitation of earlier barcoded viral approaches.^5^^,^^6^^,^^7^ Specifically, it describes one-step production of high-diversity EnvA-pseudotyped barcoded rabies virus directly from cDNA plasmids, avoiding more complex amplification-based procedures that can reduce library complexity or increase contamination risk. The workflow is implemented for both SAD-B19ΔG and CVS-N2cΔG strains and is coupled to genetically targeted starter-cell definition using TVA/N2cG helper viruses, making the method adaptable across brain regions and cell types.

Additionally, the protocol innovates upon the downstream processing of small brain tissue samples. It introduces a streamlined tissue digestion method specifically optimized to maximize single-cell recovery by omitting standard debris removal steps. This optimization ensures the successful integration of high-throughput brain-wide input mapping with single-cell transcriptomic profiling. By detailing concurrent procedures for preparing single-cell suspensions from starter regions and bulk RNA from input regions, the workflow facilitates robust barcode amplicon library construction. Together, these innovations enable researchers to accurately correlate local neural connectivity and long-range inputs with specific transcriptomic cell types.

### Institutional permissions

It is crucial to note that working with recombinant rabies virus and animal models necessitates strict adherence to safety guidelines and institutional approvals. All viral production and handling must be conducted in designated Biosafety Level 2 (BSL-2) facilities using appropriate personal protective equipment.

### Preparation of barcoded plasmids

**Timing: 1 week**

1. Assembly barcodes to rabies cDNA plasmid.
  a. In a 1.5-mL microcentrifuge tube, prepare the following reaction mixture: 4 μg of rabies virus cDNA plasmid, 10× QuickCut Buffer 10 μL, QuickCut sfiI 5 μL and Nuclease-free (ultra-pure) water to 100 μL total volume.
  b. Mix gently by pipetting or flicking the tube. Briefly spin down to collect the liquid.
  c. Seal the tube with parafilm and incubate at 50°C for 3 h.
  d. Add 50 μL of DNAClean beads to the digestion reaction. Mix thoroughly by pipetting or gentle vertexing.
  e. Purify the linearized plasmid following the manufacturer’s guidelines, typical steps include binding, magnetic separation, washing with 80% ethanol, and elution.
  f. In a 1.5-mL microcentrifuge tube, prepare the following reaction mixture: 2.5 μg purified plasmid, 2.5 μL 10 μM ssDNA (containing the random barcode sequences), 2× NEBuilder® HiFi DNA Assembly Master Mix 50 μL and Nuclease-free (ultra-pure) water to 100 μL total volume.
    ***Note:*** Ensure that the 5′ end of the ssDNA has a 15–30 bp overlap with the 5′ end of the linearized plasmid, and the 3′ end of the ssDNA has a 15–30 bp overlap with the 3′ end of the linearized plasmid. The random barcode sequence should be located between these two overlap regions.
  g. Seal the tube with parafilm and incubate at 50°C for 15–17 h.
  h. Transform 100 ng of the assembly product into chemically competent or electrocompetent E. coli. Select 10 colonies from the resulting plates for Sanger sequencing to verify barcode insertion.
2. Prepare the low-salt LB culture plates.
  a. Weigh 23 g Tryptone, 11.5 g Yeast Extract, 11.5 g NaCl, and 34.5 g agar, and add them into a 250 mL glass bottle.
  b. Mix the contents well by inverting the bottle at least 10 times.
  c. Divide the powder mixture equally into three 1 L glass bottles, 24.5 g per bottle.
  d. Add 700 mL Milli-Q water to each bottle and mix by gentle inversion.
  e. Sterilize the bottles in an autoclave and allow them to cool to ∼40°C before proceeding.
  f. Add ampicillin stock to each bottle to reach a final concentration of 100 μg/mL (0.7 mL of 100 mg/mL stock per 700 mL medium) and mix well.
  g. Place thirty 15 cm plates on a clean bench.
  h. Pour approximately 70 mL of LB medium into each plate.
  i. Allow the plates to fully solidify before use.

***Note:*** To prevent premature solidification of the medium, the bottles can be kept in a 40°C water bath before pouring.

3. Bacterial transformation and plasmid library preparation.
  a. Prepare the electroporation mixture in a 1 mm cuvette containing 100 ng purified vector DNA and 25 μL Electrocompetent Cells.
  b. Electroporate use: 10 μF, 600 Ω, 1800 V.
  c. Immediately add 975 μL Recovery Medium directly into the cuvette.
  d. Transfer the entire volume to a culture tube. Incubate at 250 rpm, 37°C, for 1 h to allow cell recovery.
  e. Plate the recovered culture onto five 15-cm low-salt LB agar plates, Incubate plates at 37°C for 15–17 h.

***Note:*** A total of six electroporation reactions and thirty 15–cm plates are used for the complete workflow. Typically, more than 30,000 clones grow per 15-cm plate.

4. Plasmid library preparation.
  a. Collect all colonies from each plate and measure the total biomass (wet weight).
  b. Purify plasmid DNA using an endotoxin-free DNA purification kit according to manufacturer’s instructions. Use one purification column per 2 g wet cell biomass (colonies scraped from plates).
  c. Quantify plasmid concentration using a NanoDrop spectrophotometer.

***Note:*** Expected yield is around 1 mg purified plasmid DNA. Ensure final plasmid concentration is >1 mg/mL for downstream applications.

### Preparation of electrotransfection buffer

**Timing: 1 h**

5. Prepare 500 mL of 1× electrotransfection buffer.
  a. Weigh 4.47 g KCl, 0.088 g KH_2_PO_4_, 0.99 g K_2_HPO_4_·3H_2_O, 2.98 g HEPES, 0.38 g EGTA, 0.55 g Na_2_ATP, 0.77 g Glutathione. 75 uL 1 M CaCl_2_, 2.5 mL 1 M MgCl_2_, adjusted PH to 7 using KOH.
  b. Pass through 0.22 μm filter and store at 4°C.
6. Prepare 50 mL of 2× electrotransfection buffer.
  a. Weigh 0.894 g KCl, 0.0176 g KH_2_PO_4_, 0.198 g K_2_HPO_4_·3H_2_O, 0.596 g HEPES, 0.076 g EGTA, 0.11 g Na_2_ATP, 0.154 g Glutathione. 15 μL 1 M CaCl_2_, 0.5 mL 1 M MgCl_2_, adjusted PH to 7 using KOH.
  b. Pass through 0.22 μm filter and store at 4°C.

## Key resources table

**Table:** 

REAGENT or RESOURCE	SOURCE	IDENTIFIER
**Bacterial and virus strains**		

SAD-B19ΔG-BFP-barcode	This paper	N/A
CVS-N2cΔG-mRuby-barcode	This paper	N/A
Electrocompetent cells	Lucigen	Cat#60242-2
AAV2/1-hsyn-DIO-H2B-mKate-P2A-TVA-F2A-N2cG	BrainVTA	N/A

**Chemicals, peptides, and recombinant proteins**		

SfiI 5′-3′ restriction endonuclease	New England Biolabs	Cat#R0123L
NEBuilder HiFi DNA Assembly Master Mix	New England Biolabs	Cat#E2621S
VAHTS 0.5 × DNA Clean Beads	Vazyme (VAHTS)	Cat#N411-01
Ampicillin (sodium salt)	BBI Life Sciences	Cat#A610028-0025
Papain	Worthington	Cat#LK003178
DNase I	Sigma-Aldrich	Cat#D4527-10KU
TRIzol reagent	Invitrogen	Cat#15596018
RNAlater stabilisation solution	Beyotime	Cat#R0118-100 mL
Maxima H-minus reverse transcriptase	Thermo Scientific	Cat#EP0752
dNTP mix	Invitrogen	Cat#R0192
RNase Out inhibitor	Invitrogen	Cat#10777019
PrimeSTAR Max DNA polymerase	TaKaRa	Cat#R045A
High-glucose DMEM	Gibco	Cat#11995081
Fetal bovine serum (FBS)	Thermo Fisher	Cat# 10099141C
Opti-MEM I reduced-serum medium	Gibco	Cat#11058021
0.25% Trypsin-EDTA	Gibco	Cat#25200072
DNA clean beads	VAHTS	Cat#N411-01
DMSO	Sigma-Aldrich	Cat#D2650
Benzonase nuclease	HaiGene	Cat#C2002-100KU
nuclease-free water	Ambion	Cat#AM9938
KCl	Sigma-Aldrich	Cat#P5405-500 g
KH_2_PO_4_	Sigma-Aldrich	Cat#529568-250GM
K_2_HPO_4_·3H_2_O	Sigma-Aldrich	Cat#529567-M
HEPES	Sigma-Aldrich	Cat# V900477-500G
Na_2_ATP	Sigma-Aldrich	Cat#A26209-10G
Glutathione	Sigma-Aldrich	Cat#PHR1359-500MG
EGTA	Sigma-Aldrich	Cat#E3889-10G
CaCl_2_ 1 M stock	teknova	Cat#C0478
MgCl_2_ 1 M stock	Invitrogen	Cat#AM9530G
NMDG	Sigma-Aldrich	Cat#M2004-1KG
HCl (36.5%–38%)	Sigma-Aldrich	Cat#H1758-100ML
NaH_2_PO_4_	Sigma-Aldrich	Cat#V900060-500G
Glucose	Sigma-Aldrich	Cat#V900392-1KG
Sodium ascorbate	Sigma-Aldrich	Cat#A4034-100G
Thiourea	Sigma-Aldrich	Cat#T7875-500G
Sodium pyruvate	Sigma-Aldrich	Cat#V900232-100G
N-Acetyl-L-cysteine (NAC)	Sigma-Aldrich	Cat#A7250-50G
MgSO_4_	Sigma-Aldrich	Cat#M7506-1Kg
Tryptone	Beyotime	Cat#ST800
Yeast Extract	Beyotime	Cat#ST968
NaCl	Sigma-Aldrich	Cat#V900058-500G
Agar	Beyotime	Cat#ST004E

**Critical commercial assays**		

Endotoxin-free plasmid maxi kit	Macherey-Nagel	Cat#740426.5
Papain	Worthington	Cat#LK003178

**Deposited data**		

Demo data and scripts	This study	Figshare: https://doi.org/10.6084/m9.figshare.31072744

**Experimental models: Cell lines**		

HEK-293 T-TVA cells	Gifted from Dr. Edward Callaway	HEK 293 T–TVA800 cells are derived from HEK 293 T cells and express TVA.^3^^,^^4^
BHK-EnvA cells	Gifted from Dr. Edward Callaway	BHK-EnvA cells are derived from BHK-21 cells and express a chimeric envelope protein consisting of the extracellular and transmembrane domains of EnvA fused to the cytoplasmic domain of rabies virus G.^3^^,^^4^

**Experimental models: Organisms/strains**		

C57BL/6 J mice, 8 weeks old, male	Charles River	Cat#219

**Oligonucleotides**		

N2c-RT-Index	CCAGTGTAAGCCGATCTGC R_8_ N_12_ GATGACCCAGCCCTCAATAG	Sangon Biotech
Barcode-linker-F	CCTACACGACGCTCTTCCGATCTG ACAATGAAACCTACGTAGTGC	Sangon Biotech
Linker1st-R	TCAGACGTGTGCTCTTCCGATCCCAGT GTAAGCCGATCTGC	Sangon Biotech
MGI-F	CCTACACGACGCTCTTCCGATCTGAACG ACATGGCTACGATCCG	Sangon Biotech
Barcode-R	TCAGACGTGTGCTCTTCCGATCTTGGCA GTTGCCAAATACAGC	Sangon Biotech
AdapterF	AATGATACGGCGACCACCGAGATCTAC ACACACTCTTTCCCTACACGACGC	Sangon Biotech
IndexR	CAAGCAGAAGACGGCATACGAGAT N_8_ GTGACTGGAGTTCAGACGTGTGCT	Sangon Biotech
CVS-BCV1 cassette	GGACGAGCTGTACAAGTAAGGTACC GGCCATTANNNACNNNGTNNNCGN NNTANNNCANNNTGNNNAGGCGGC CCTATTGAGGGCTGGGTCATCTAAGC	Sangon Biotech
Spike-In template F	TAATACGACTCACTATAGGGAGTGAC AATGAAACCTACGTAGTGCAAAGAGA AGTGGCAGTTGCCAAATACAGCAACC TTGGTGGTGGCATGGACGAGCTGTAC AAGTAAGGTACCGGCCAT	Sangon Biotech
Spike-In template R	TTTTTTTCTCGACTGAAATGCTTAGAT GACCCAGCCCTCAATAGGGCCGCCT CGGCCNNNTGNNNACNNNATNNNG CNNNTGNNNACNNNGGCCGTAATG GCCGGTACCTTA	Sangon Biotech

**Recombinant DNA**		

pSADΔG-BFP-BC	This study	N/A
pCAG-T7pol	Lei Jin Lab	Addgene, 59926
pCAG-B19N	Lei Jin Lab	Addgene, 59924
pCAG-B19P	Lei Jin Lab	Addgene, 59925
pCAG-B19L	Lei Jin Lab	Addgene, 59922
pCAG-B19G	Lei Jin Lab	Addgene, 59921
pCAG-EnvARGCD	Lei Jin Lab	N/A
pCAG-TVA	This study	N/A
pCVS-N2cΔG-mRuby-BC	This study	N/A

**Software and algorithms**		

R4.2.2	The R Project for Statistical Computing	https://www.r-project.org/
ShortRead (Bioconductor)	v 1.64.0	https://bioconductor.org/packages/ShortRead/
Biostrings (Bioconductor)	V 2.74.1	https://code.bioconductor.org/browse/Biostrings/
dplyr	V 1.1.4	https://dplyr.tidyverse.org/

**Other**		

15 cm plates	JET	Cat#TCD010150
10 cm dishes	Corning	Cat#430293
0.22 μm syringe filters	BIOFIL	Cat#FPE204025
4 mm electroporation cuvettes	BIORAD	Cat#1652081
Gene Pulser Xcell Electroporation Systems	BIORAD	Cat#1652661
0.45 μm syringe filters	BIOFIL	Cat#FPE404030
0.45 μm vacuum bottle filters	BIOFIL	Cat#FPE404250
200 μL low-binding tubes	axygen	Cat#AXY-PCR-02-L-C
SW32Ti rotor	Beckman Coulter	Cat#369694
15 cm plates	Corning	Cat#354551
50 mL tube	Corning	Cat#430828
Open top centrifuge tubes	Beckman	Cat#344058
Titanium Micro Point	FST	Cat#10165-11
Ultracentrifuge	Beckman Coulter	CAT#Optima XE-90
38.5 mL, Open-Top Thinwall Polypropylene Centrifuge Tube	Beckman Coulter	CAT#326823

## Materials and equipment

**Table:** Low-salt LB bacterial culture plates

Reagent	Final concentration	Amount
Tryptone	1.10%	11.0 g
Yeast Extract	0.55%	5.5 g
NaCl	0.55%	5.5 g
Agar	1.64%	16.4 g
Milli-Q water	N/A	Bring total volume to 1 L
Ampicillin (100 mg/mL stock)	100 μg/mL final	1 mL

Add ampicillin stock only after the medium has cooled to ∼40°C to prevent heat-induced degradation. Store at 4°C for up to 3 months.

**Table:** Electrotransfection buffer

Reagent	Final concentration	Amount for 500 mL
KCl	120 mM	4.47 g
KH_2_PO_4_	1.3 mM	0.088 g
K_2_HPO_4_·3H_2_O	8.7 mM	0.99 g
HEPES	25 mM	2.98 g
EGTA	2 mM	0.38 g
Na_2_ATP	2 mM	0.55 g
Glutathione	5 mM	0.77 g
CaCl_2_ (from 1 M stock)	0.15 mM	75 μL
MgCl_2_ (from 1 M stock)	5 mM	2.5 mL
Milli-Q water	N/A	To 500 mL total volume

Adjust pH to 7.0 with KOH after dissolving all components (before bringing to final volume). Filter-sterilize through a 0.22 μm filter. Store at 4°C for up to 3 months.

**Table:** Brain slice preparation buffer

Reagent	Final concentration (mM)	Amount for 1 L
NMDG	93 mM	18.16 g
HCl (36.5–38%)	— (pH adjust)	∼7 mL (adjust pH to 7.4–7.5)
KCl	2.5 mM	0.19 g
NaH_2_PO_4_	1.2 mM	0.17 g
NaHCO_3_	30 mM	2.52 g
HEPES	20 mM	4.77 g
Glucose	25 mM	4.51 g
Sodium ascorbate	5 mM	0.99 g
Thiourea	2 mM	0.15 g
Sodium pyruvate	3 mM	0.33 g
N-Acetyl-L-cysteine (NAC)	12 mM	1.96 g
MgSO_4_	10 mM	1.203 g
CaCl_2_ (from 1 M stock)	0.5 mM	500 μL of 1 M stock
Milli-Q water	—	Bring to 1 L total volume

Final osmolarity: 300–310 mOsm. Adjust pH to 7.4–7.5 with 10 HCl (∼7 mL per 1 L, typical). Store at 4°C for up to 1 week.

**Table:** Single cell digestion working solution

Reagent	Final concentration	Amount for 2.5 mL
Oxygenated NMDG/HEPES solution	—	2.5 mL
Papain	20 U/mL	50 U total
DNase I	1000 U/mL	2500 U total

Activate the solution at 37°C for 10 min before using. Prepare the solution just before use.

**Table:** Cell resuspension buffer

Reagent	Final concentration	Amount for 25 mL
Oxygenated NMDG/HEPES solution	—	24.5 mL
FBS	2% (v/v)	0.5 mL
DNase I	10 U/mL	250 U total

Filter-sterilize through a 0.22 μm filter. Prepare the solution just before use.

**Table:** Sample run buffer

Reagent	Final concentration	Amount for 25 mL
Oxygenated NMDG/HEPES solution	—	24.5 mL
FBS	2% (v/v)	0.5 mL

Filter-sterilize through a 0.22 μm filter. Prepare the solution just before use.

## Step-by-step method details

### One-step packaging of barcoded rabies virus

**Timing: 2 weeks**

**Figure 1:**
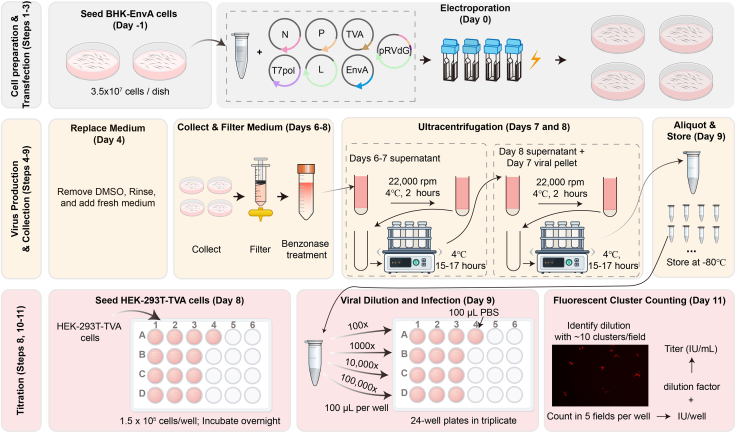
Workflow for rabies virus production, purification, and titration This figure summarizes the workflow for rabies virus production and titration. BHK-EnvA cells are seeded on day −1 and electroporated on day 0 with the plasmids required for virus rescue and production. After medium replacement on day 4, viral supernatant is collected on days 6–8, filtered, treated with Benzonase, and concentrated by ultracentrifugation over 2 days. The purified virus is then aliquoted and stored at −80°C on day 9. For titration, HEK-293 T-TVA cells are seeded on day 8 and infected on day 9 with serial virus dilutions. Fluorescent clusters are counted on day 11 to identify the appropriate dilution and calculate viral titer. Step numbers shown on the left correspond to the detailed protocol steps.

In this step, we describe how to directly package EnvA pseudotyped rabies virus from RV cDNA plasmids (Figure 1).

1. Cell preparation (Day 0).
  a. Culture BHK-EnvA cells in high-glucose DMEM supplemented with 10% FBS.
  b. Day −1: Seed cells into two 15-cm dishes, 3.5 × 10^7^ cells per plate.
  c. Day 0: ∼14 h after cell passage, detach cells using 0.25% trypsin-EDTA.
  d. Pellet cells at 200 × *g* for 5 min at 4°C in DMEM + 10% FBS.
  e. Discard the supernatant and resuspend the pellet in 1 mL ice-cold electroporation solution.
  f. Count cells.
  g. Transfer 6 × 10^7^ cells into a fresh tube and rinse with 7 mL ice-cold electroporation solution.
2. Plasmid Mixture Preparation.
  a. Prepare the plasmid mixture described in Table 1.

**Table 1:** Component of plasmid mixture

Plasmid	Amount (μg)
pRVΔG	217 μg
pCAG-T7pol	55.5 μg
pCAG-B19N	85 μg
pCAG-B19P	43.8 μg
pCAG-B19L	37.5 μg
pCAG-EnvARGCD	45 μg
pCAG-TVA	23.5 μg
  b. Combine plasmids and mix with an equal volume of 2× electroporation solution.
  c. Adjust the mixture plus cells to a final volume of 800 μL with ice-cold electroporation solution.
  d. Add 10 μL of DMSO to the mixture and gently pipette to mix thoroughly.
3. Electroporation.
  a. Aliquot 800 μL of the cell-plasmid suspension into four 4-mm electroporation cuvettes.
    **CRITICAL:** Avoid introducing bubbles. If bubbles form in the cuvettes, carefully remove them using a 200-μL pipette tip.
  b. Gently shake the cuvettes to mix the cells thoroughly, then wipe the metal surfaces dry with dust free tissues before electroporation.
  c. Electroporate using a Bio-Rad system with the following settings: 250 V, 10 pulses, 5 ms duration each pulse, and a 1 s inter pulse interval.
    **CRITICAL:** Reduce the voltage or pulse duration if excessive cell death is observed after transfection (Figures 2A and 2B). Preliminary experiments to optimize voltage and pulse duration are recommended.

**Figure 2:**
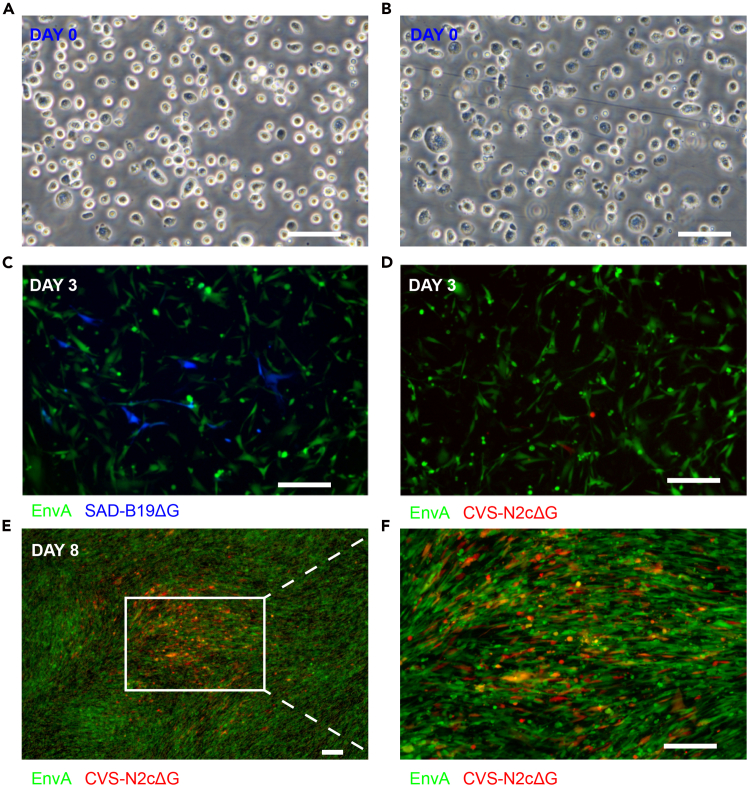
Expected results under microscopy (A) Representative image of cells expected to exhibit healthy growth after transfection. (B) Representative image of cells expected to exhibit poor growth after transfection. (C) Representative SAD-B19ΔG rescued cells at day 3. (D) Representative CVS-N2cΔG rescued cells at day 3. (E and F) Clusters of CVS-N2cΔG rescued and infected cells at day 8. Scale bar, 200 μm.
  d. Prepare four 15-cm dishes, each containing 25 mL high-glucose DMEM + 10% FBS + 1.25% DMSO (one dish per cuvette).
  e. For each cuvette, add 200 μL of medium to the cuvette and gently shake to resuspend the cells.
  f. Transfer the 200 μL cell suspension to a 15-cm dish containing 25 mL high-glucose DMEM + 10% FBS + 1.25% DMSO.
  g. Add an additional 200 μL of medium to the cuvette, repeat the resuspension, and transfer all remaining medium to the dish.
    ***Note:*** Viscous cell aggregates often form after transfection. Adding fresh medium helps transfer the cells without aspirating the aggregates.
    ***Note:*** Using SAD-B19ΔG, the expected transfection efficiency is ∼1/500 (∼0.2%), typically yielding ∼10 fluorescent-positive cells within the 10× objective field of view (3.8 mm² FOV) or clusters at 3 days post-transfection. In comparison, CVS-N2cΔG is approximately 5–10-fold less efficient than SAD-B19ΔG, typically yielding ∼1–2 fluorescent-positive cells or clusters at 3 days post-transfection (Figures 2C and 2D). Notably, initially labeled single cells or small clusters are expected to grow into larger clusters during the subsequent incubation period (Figures 2E and 2F). We also tested commonly used chemical transfection reagents, including Polyethylenimine (PEI) and Lipofectamine 2000 (Lipo2000), but both showed lower transfection efficiency.^1^
4. Replace medium (Day 4).
  a. Remove medium, rinse each plate with 10 mL DPBS to remove DMSO.
  b. Replace with 25 mL fresh medium containing 25 mL high-glucose DMEM + 10% FBS.

***Note:*** We did not collect the medium on those days due to the presence of 1.25% DMSO.

5. Collect medium (Day 6).
  a. Collect 12 mL of medium from each plate.
  b. Replace with 12.5 mL fresh medium.
  c. Filter the collected medium through a 0.45 μm filter and store at 4°C.
6. Collect medium (Day 7).
  a. Collect 12 mL of medium from each plate.
  b. Replace with 12.5 mL fresh medium.
  c. Filter the collected medium through a 0.45 μm filter and combined with the medium collected at day 6.
  d. Treat pooled medium with 30 U/mL benzonase nuclease at 37°C for 30 min. Mixing the medium every 10 min.
  e. Prepare three ultracentrifuge tubes with 5 mL of 20% (w/v) sucrose in PBS.
  f. In each ultracentrifuge tube, carefully overlay 33 mL of the virus-containing medium onto the sucrose layer, avoiding disruption of the clear demarcation line. If the remaining virus-containing medium is less than 33 mL, top up to 33 mL with fresh medium.
    ***Optional:*** Add 10 mL of virus-containing medium to the ultracentrifuge tube, then underlay with 5 mL of 20% (w/v) sucrose in PBS using a 5 mL serological pipette (maximum usable volume of 8 mL with a stretched tip). It is recommended to draw up 6.5 mL of 20% sucrose and dispense only 5 mL slowly to minimize bubble formation.
  g. Balance the buckets and ultracentrifuge at 22,000 rpm (82,700 × *g*), 4°C, 2 h using a Beckman SW32Ti rotor.
  h. Remove supernatant, invert the ultracentrifuge tubes onto dust-free tissue and allow them to drain for 5 min.
  i. Remove any remaining liquid on tube walls using vacuum pump.
  j. For each ultracentrifuge tube, resuspend viral pellets in 200 μL DPBS by gently pipette.
  k. Return the tubes to the centrifuge buckets and gently shake them at 4°C for 15–17 h.
7. Collect and ultracentrifuge (Day 8).
  a. Collect remaining medium.
  b. Filter through a 0.45 μm filter.
  c. Treat with 30 U/mL benzonase nuclease at 37°C for 30 min.
  d. Prepare tubes with 5 mL 20% sucrose cushion.
  e. Overlay the medium and the previously concentrated virus from Day 7.
    **CRITICAL:** Overlaying the Day 7 concentrate onto the next ultracentrifugation run is used only to increase final titer for CVS-N2cΔG when the expected yield is low. Because a second ultracentrifugation subjects Day 6–7 virions to additional mechanical stress and repeat pelleting (which may reduce infectivity), do not perform this overlay unless the final titer is expected to be < 2 × 10^7^. For SAD-B19ΔG, which typically reaches higher titers, collect the Day 7 concentrate separately and combine it with the Day 8 concentrate after ultracentrifugation to avoid double pelleting.
  f. Ultracentrifuge again at 22,000 rpm (82,700 × *g*), 4°C, 2 h.
  g. Remove supernatant.
  h. Inverting the ultracentrifuge tubes onto dust-free tissue and allowing them to drain for 5 min. Remove any remaining liquid on tube walls using vacuum pump.
  i. For each ultracentrifuge tube, resuspend viral pellets in 20 μL DPBS by gently pipette.
  j. Return the tubes to the centrifuge buckets and gently shake them on a rocking shaker at 4°C for 15–17 h.
8. Cell Seeding (Day 8).
  a. Seed 1.5 × 10^5^ HEK-293 T-TVA cells per well into 13 wells of a 24-well plate.
  b. Add medium to a final volume of 500 μL per well.
  c. Incubate for 17–19 h.
9. Collect and ultracentrifuge (Day 9).
  a. Combine all resuspended virus volumes into a single tube and mix gently by pipetting.
  b. Aliquot into low-protein-binding tubes at 2 to 3 μL per tube.
  c. Store at −80°C.
10. Viral dilution and infection (Day 9).
  a. Mix 3.5 μL viral stock with 350 μL medium to prepare a 100× dilution.
  b. Transfer 50 μL of the 100× dilution into 450 μL medium to prepare a 1000× dilution.
  c. Transfer 50 μL of the 1000× dilution into 450 μL medium to prepare a 10,000× dilution.
  d. Transfer 50 μL of the 10,000× dilution into 450 μL medium to prepare a 100,000× dilution.
  e. Add 100 μL of each dilution (100×, 1000×, 10,000×, and 100,000×) to three replicate wells per dilution.
  f. Gently dispense virus to avoid disturbing the cell monolayer.
  g. Return the plate to the incubator and culture.
11. Fluorescent Cluster Counting (Day 11).
  a. Identify the dilution producing 1–10 fluorescent clusters per field under a 10× objective.
  b. For that dilution, count clusters in five fields per well (up, down, left, right, center), each covering 3.8 mm^2^ (the field of view of our 10× objective).
    ***Note:*** Treat adjacent fluorescent cell clusters as one infection unit due to cell division between Day 9 and Day 11.
  c. Collect cluster counts from 15 fields (3 wells × 5 fields).
  d. Remove outliers using a boxplot-based method.
  e. Average the remaining counts to obtain c.
  f. The growth area of a 24-well plate well is 198 mm^2^, equivalent to 52.1 fields/well.
  g. The viral titer (IU/mL) is calculated as: $Virus\;Titer\;\left(\frac{IU}{mL}\right)=521\cdot c\cdot x$
  h. where c = mean cluster count per field, x = dilution factor used, 521 = 52.1 fields × 10 (to convert 100 μL infection volume to 1 mL).
    ***Note:*** Adjust the field of view (FOV) value according to the manufacturer’s specifications for your microscope.
    ***Note:*** This fluorescent cluster–counting method quantifies infectious units (IU/mL), analogous to FACS-based titration that measures the fraction of fluorescent (infected) cells. For results comparable to FACS, perform infections in the low-MOI range (e.g., choose dilutions yielding sparse, well-separated clusters) so that each fluorescent cluster is seeded by a single initial infection event. Because microscope optics, exposure, and counting thresholds can vary across setups, users may optionally perform a one-time side-by-side titration of the same viral stock by FACS to confirm agreement and establish local counting parameters.

### Injection of helper virus and barcoded rabies virus

**Timing: 4 weeks**

In this step, we describe the procedure for injecting the helper virus and, three weeks later, the barcoded rabies virus into the target brain region.

12. AAV injection (Day 0).
  a. Load 15 nL viral mixture containing AAV2/1-hSyn-DIO-H2B-mKate-P2A-TVA-F2A-N2cG (1 × 10^12^ vg/mL) and AAV2/9-hSyn-Cre-NLS (6.25 × 10^9^ vg/mL).
    ***Note:*** For future validation of co-labeling with CVS-N2cΔG-mRuby-barcode, AAV2-hSyn-DIO-H2B-EGFP-P2A-TVA-F2A-N2cG provides a more convenient option.
  b. Inject the mixture into the target brain region slowly using a stereotaxic apparatus of adult C57BL/6 J mice (2–3 months old) at a rate of ∼3 nL/min.
    ***Note:*** The coordinates of −1.65 mm AP, 0.33 mm ML, and −1.45 mm DV target the mPFC in our hands. These coordinates should be validated using dye and adjusted as needed to correct any deviations before performing formal injections.
  c. After injection, wait 5 min before slowly withdrawing the injection electrode from the brain.
    **CRITICAL:** The titer and volume used here typically infect ∼1,500 cells in the mouse mPFC. The viral mixture volume and the titer of AAV2/9-hSyn-Cre-NLS should be carefully optimized to achieve the desired number of infected cells.
13. RV Injection (3 weeks later).
  a. Load 200 nL of CVS-N2cΔG-mRuby-barcode (2 × 10^7^ IU/mL).
  b. Inject the rabies virus into the same coordinates, with a faster rate of ∼20 nL/min.
  c. After injection, wait 10 min before slowly withdrawing the injection electrode from the brain.

***Note:*** During surgery, maintain mice under 1.5% isoflurane anesthesia and keep body temperature at 37°C. After the injection, suture the scalp and apply lincomycin hydrochloride and lidocaine hydrochloride gel to reduce pain and prevent infection. Following the procedure, place the mouse on a heating pad and monitor closely until it has fully recovered from anesthesia.

14. Allow RV expression and trans-synaptic spread to proceed for 7 to 8 days before downstream processing.

### Separation of target regions from brain slices

**Timing: 1 day**

This section outlines the procedures for processing the starter and input regions. The input for this step consists of animals that have received barcoded rabies virus injections. The outputs are a single-cell suspension from the starter region, suitable for commercial single-cell library preparation platforms such as 10× Genomics V3 or DNBC4 V3, and total RNA from the input regions for downstream processing. Before starting, clean the bench and all tools with an RNase decontamination solution, and wear appropriate personal protective equipment throughout the procedure.

15. Brain perfusion and sectioning.
  a. Transcranially perfuse the mouse with ice-cold, oxygenated NMDG/HEPES solution using a peristaltic pump, continuing the perfusion until the fluid exiting the animal becomes completely clear.
  b. Secure the brain onto the vibratome stage using quick-dry glue, then section it into 300-μm coronal slices in ice-cold, oxygenated NMDG/HEPES solution using a vibratome set to a slow cutting speed of 0.04 mm/s.
  c. Transfer each brain slice using a wide-mouthed glass straw into 34°C oxygenated NMDG/HEPES solution and allow the slices to recover for 12–15 min.
  d. Transfer each brain slice into oxygenated NMDG/HEPES solution at 20°C–24°C and keep them there until further processing.

**Figure 3:**
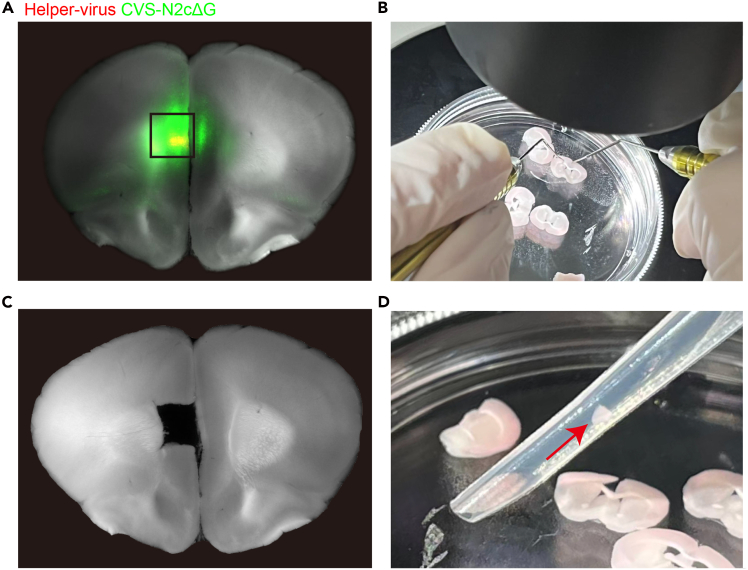
Visualization of the starter region dissection for digestion (A) Verify colocalization of the helper virus and rabies virus, and define the boundary for dissection. (B) Under a dissection microscope, isolate the target region using a pair of titanium micro points. (C) Document the boundary of the excised tissue. (D) Transfer the tissue using a Pasteur pipette.
16. Identification and dissection of target regions.
  a. Transfer a brain slice to a well of six well plate with ice cold oxygenated NMDG/HEPES solution.
  b. Under a fluorescent dissecting microscope, examine each slice to assess rabies virus expression and verify co-labeling with the helper virus (Figure 3A).
    **CRITICAL:** Ensure that the entire helper virus-expressing region is fully covered by rabies virus fluorescence. If any portion of the helper virus-expressing region remains uninfected by the rabies virus, the animal should be excluded for future processing.
  c. Capture images of the entire brain slice in both bright-field and fluorescence modes.
  d. Under fluorescence, carefully dissect the target regions using a pair of titanium micro-pointers (Figure 3B).
  e. After dissection, take a bright-field image of the entire slice to document the boundaries of the removed tissue (Figure 3C).
  f. For tissue intended for bulk barcode sequencing, transfer the dissected regions into a 1.5-mL centrifuge tube containing 1 mL RNAlater.
    ***Pause point:*** The target regions can be stored for a week at 4°C.
    ***Note:*** Tissues originating from the same anatomical structure may be pooled into a single tube if slice-to-slice differences within that structure are not relevant to the analysis.
    ***Note:*** It is recommended to perform digestion of the starter regions in parallel with the dissection of input regions.
17. Tissue digestion.
  a. Prepare 2.5 mL of papain working solution in a 15 mL centrifuge tube and activate it at 37°C for 10 min.
  b. For tissue intended for single-cell RNA sequencing, gently transfer the dissected regions into the papain working solution using a Pasteur pipette (Figure 3D).
  c. Seal the tube cap with parafilm, then incubate the tissue in the digestion solution at 37°C for 30 min on a rotating shaker.
    ***Note:*** Ensure that the tissue is freely floating in the solution on the rotating shaker, and not adhered to the tube walls, where it would be inaccessible to the solution.
    **CRITICAL:** Digestion time may vary across brain regions. A 30-min digestion is generally suitable for mouse cortex, but optimization via pilot experiments is strongly recommended.
  d. Pre-cool the centrifuge to 4°C, then centrifuge the digested tissue at 300 × *g* for 5 min.
  e. Carefully remove the supernatant and replace it with 2 mL of cell resuspension buffer.
  f. Keep the tube on ice. Attach the glass pipette to a rubber bulb, then gently dissociate the tissue by repeatedly passing a glass pipette with a 300-μm tip until no significant resistance is detected (typically ∼10 passes).
  g. Further dissociate the tissue using a 200-μm tip glass straw until no visible tissue clumps remain (typically <20 passes).
    ***Note:*** Extend the digestion time and cut the tissue into smaller pieces if clumps remain after 20 passes, as this will greatly reduce the number of recovered cells.
  h. Filter the resulting cell suspension through a 40-μm cell strainer pre-rinsed with sample run buffer.
  i. Adjust the filtered suspension to a final volume of 10 mL with additional sample run buffer.
  j. Centrifuge at 300 × *g* for 5 min at 4°C.
  k. Carefully discard the supernatant without disturbing the pellet, then resuspend the cells in 40 μL of sample run buffer.
    **CRITICAL:** Ensure that the sampling volume and buffer composition are within the limits specified by the manufacturer for downstream single-cell library preparation.
18. Assess cell concentration and viability by mixing 4 μL of the cell suspension with 4 μL of trypan blue. Use the resulting single-cell suspension for single-cell sequencing.

***Note:*** This streamlined digestion method was optimized for small tissue input, and debris removal was omitted to maximize cell recovery. However, if tissue input is relatively large and substantial debris is generated using this method, incorporating an additional debris removal step is recommended.

19. RNA Extraction of input regions.
  a. Briefly centrifuge the tube, remove RNAlater and replace it with 1 mL TRIzol reagent.
  b. Add two steel beads to each tube.
  c. Homogenize the tissue at 200 Hz for 120 s at 4°C using a vibrating tissue homogenizer.
  d. Briefly centrifuge the tube, then transfer the solution to a new tube.
  e. Processed the RNA extraction according to manufacturer’s instructions.
  f. Storing the samples at −80°C.

### Barcode amplicon library preparation

**Timing: 2 days**

**Figure 4:**
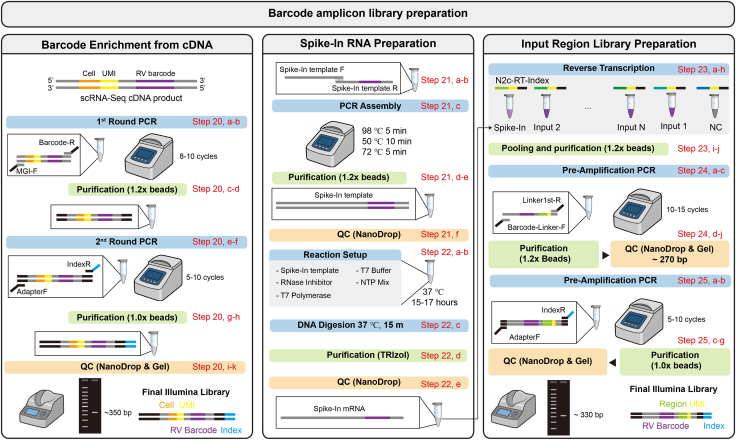
Workflow for barcode amplicon library preparation This figure summarizes the workflow for barcode amplicon library preparation in this part. The left panel shows barcode enrichment from scRNA-seq cDNA by two rounds of PCR, followed by bead purification and quality control to generate the final Illumina barcode library. The middle panel shows spike-in RNA preparation, including spike-in template assembly, purification, in vitro transcription, DNA digestion, RNA purification, and quality control. The right panel shows input region library preparation, including reverse transcription, pooling and purification, pre-amplification PCR, and quality control to generate the final Illumina input-region library. Step numbers in red correspond to the detailed protocol steps.

This step describes the preparation of the RV-barcode library (Figure 4). The inputs are the cDNA products from the commercial single-cell platform or the total RNA from the input regions. The outputs are Illumina libraries of RV-barcode amplicons from both the starter and input regions.

20. Enrichment of barcodes from cDNA product.
  a. In a PCR tube, prepare a 100 μL reaction containing 60 ng cDNA, as described in Table 2.

**Table 2:** PCR mixture composition 1

	Volume per reaction	Stock concentration	Final concentration
PrimeSTAR Max	50 μL	-	-
MGI-F	10 μL	10 μM/μL	1 μM/μL
Barcode-R	10 μL	10 μM/μL	1 μM/μL
cDNA Product	x μL (60 ng)	-	-
Ultrapure water	30 - x μL	-	-
  b. Perform the first-round PCR using the program described in Table 3.
    ***Note:*** The number of PCR cycles depends on the abundance of rabies virus in the cDNA; 8–10 cycles are typically sufficient. Additional cycles can be performed if the target sequences are not detected in the final PCR product. However, cycling should remain moderate to avoid introducing biases.

**Table 3:** PCR program 1

Steps	Temperature	Time	Cycles
Initial Denaturation	98°C	5 min	1
Denaturation	98°C	30 s	8–10 cycles
Annealing	58°C	30 s	-
Extension	72°C	1 min	-
Final extension	72°C	5 min	1
Hold	12°C	Forever
  c. Purify the first-round PCR products using 1.2× DNA clean beads according to the manufacturer’s instructions.
    ***Note:*** PCR may lose small amounts of volume due to evaporation. To ensure accurate bead-to-sample ratio, set the pipette to 100 μL. If the volume is less than 100 μL, add ddH_2_O to bring it up to 100 μL.
  d. Elute the purified product in 20 μL ultrapure water.
  e. In a new PCR tube, prepare a 100 μL second-round PCR reaction according to Table 4.

**Table 4:** PCR mixture composition 2

	Volume per reaction	Stock concentration	Final concentration
PrimeSTAR Max	50 μL	-	-
AdapterF	10 μL	10 μM/μL	1 μM/μL
IndexR	10 μL	10 μM/μL	1 μM/μL
PCR product	20 μL	-	-
Ultrapure water	10 μL	-	-
  f. Perform the second-round PCR using the program described in Table 5.

**Table 5:** PCR program 2

Steps	Temperature	Time	Cycles
Initial Denaturation	98°C	5 min	1
Denaturation	98°C	30 s	5–10 cycles
Annealing	52°C	30 s	-
Extension	72°C	1 min	-
Final extension	72°C	5 min	1
Hold	12°C	Forever
  g. Purify the second-round PCR products using 1.0× DNA clean beads according to the manufacturer’s instructions.
  h. Elute the final PCR product in 20 μL ultrapure water.
  i. Clean the NanoDrop pedestal with ddH_2_O, measure a baseline using 1 μL ddH_2_O, then determine DNA concentration using 1 μL purified product.
  j. Run gel electrophoresis using 4 μL purified product.
  k. Expected result is a clear band at ∼350 bp and no primer-dimer (∼100 bp).
21. Assembly of Template DNA for Spike-In mRNA.
  a. Synthesize two complementary single-stranded DNA oligos containing a 15-bp overlapping region, with a T7 promoter placed upstream of the forward sequence.
  b. Prepare a 20 μL reaction according to Table 6.

**Table 6:** T7 template sequence assembly mixture

	Volume per reaction	Stock concentration	Final concentration
PrimeSTAR Max	10 μL	-	-
Spike-In template F	1 μL	100 μM/μL	10 μM/μL
Spike-In template R	1 μL	100 μM/μL	10 μM/μL
Ultrapure water	8 μL	-	-
  c. Place the tube in a PCR thermocycler and run the program described in Table 7.

**Table 7:** T7 template sequence assembly PCR

Steps	Temperature	Time	Cycles
Initial Denaturation	98°C	5 min	1
Annealing	50°C	10 min	1
Final extension	72°C	5 min	1
Hold	12°C	Forever
  d. Purify the PCR product using 1.2× DNA clean beads according to the manufacturer’s instructions.
  e. Elute the purified DNA in 20 μL of ultrapure water.
  f. Measure the DNA concentration using a NanoDrop spectrophotometer.
22. *In Vitro* Transcription of Spike-In mRNA.
  a. Prepare a 20 μL *in vitro* transcription reaction according to Table 8.

**Table 8:** T7 invitro assembly mixture

Reagent	Amount
T7 Reaction Buffer (10×)	2 μL
NTP Mix	8 μL
Template DNA	X μL
RNase Inhibitor	0.5 μL
T7 RNA Polymerase	2 μL
Water, nuclease-free	7.5-X
Total	20 μl
  b. Incubate the reaction at 37°C for 15–17 h in a thermocycler.
  c. Add 80 μL nuclease-free water and 1 μL DNase I, mix gently, and incubate at 37°C for 15 min to digest template DNA.
  d. Purify RNA using TRIzol following the manufacturer’s protocol.
  e. Measure RNA concentration using a NanoDrop spectrophotometer.
23. Reverse translates the cDNA of barcodes region from input mRNA.
  a. For each input region (including the negative control and spike-in control), assemble the following reaction in a sterile, nuclease-free tube on ice, adding the components in the order listed in Table 9.

**Table 9:** RT-PCR mixture part 1

Reagent	Amount
RNA	2 μL
N2c-RT-Index (10 μM/μL)	1 μL
dNTP Mix, 10 mM each	1 μL
Water, nuclease-free	To 14.5 μL
  b. Mix gently, centrifuge briefly.
  c. Incubate at 65°C for 5 min.
  d. Immediately chill on ice, briefly centrifuge, and keep the tube on ice.
  e. Add the following components to the reaction tube in the order listed in Table 10.

**Table 10:** RT-PCR mixture part 2

Reagent	Amount
5× RT Buffer	4 μL
RNase Inhibitor	0.5 μL
Maxima H Minus Reverse Transcriptase	0.5 μL
Total volume	20 μL
  f. Mix gently and centrifuge briefly.
  g. Incubate for 20 min at 50°C.
  h. Terminate the reaction by heating at 85°C for 5 min.
  i. Collecting 2 μL of each cDNA product and mixing in a new tube. Store the remaining cDNA at −80°C.
  j. Purify the mixed cDNA using 1.2× DNA clean beads according to the manufacturer’s instructions.

***Pause point:*** The samples can be stored for several days at −20°C.

24. Pre-Amplification of input cDNA.
  a. In each tube, combine the components listed in Table 11.

**Table 11:** PCR mixture composition 3

	Volume per reaction	Stock concentration	Final concentration
PrimeSTAR max	25 μL	-	-
RT-PCR purified product	10 μL	-	-
Barcode-linker-F	5 μL	10 μM/μL	1 μM/μL
Linker1st-R	5 μL	10 μM/μL	1 μM/μL
Ultrapure water	5 μL	-	-
  b. Mix by gentle inversion and briefly centrifuge.
  c. Perform PCR amplification using the program described in Table 12.

**Table 12:** PCR program 3

Steps	Temperature	Time	Cycles
Initial Denaturation	98°C	5 min	1
Denaturation	98°C	30 s	10–15 cycles
Annealing	58°C	30 s	-
Extension	72°C	1 min	-
Final extension	72°C	5 min	1
Hold	12°C	Forever
  d. Remove 4 μL from each reaction for agarose gel electrophoresis.
  e. Adjust the total reaction volume to 16 μL.
    ***Note:*** PCR may lose small amounts of volume due to evaporation. To ensure accurate bead-to-sample ratio, set the pipette to 16 μL. If the volume is less than 16 μL, add ddH_2_O to bring it up to 16 μL.
  f. Purify the PCR product using 1.2× DNA beads, adding 19.2 μL beads per tube.
  g. Elute purified DNA in 20 μL Ultrapure water.
  h. Clean the NanoDrop pedestal with ddH_2_O, measure a baseline using 1 μL ddH_2_O, then determine DNA concentration using 1 μL purified product.
  i. Run gel electrophoresis using 4 μL purified product and 4 μL unpurified product.
  j. Expected result is a correct band at ∼270 bp and no primer-dimer (∼100 bp).
    ***Pause point:*** The samples can be stored for several days at 4°C.
25. Indexing PCR of input products.
  a. For each reaction, combine the components listed in Table 13.

**Table 13:** PCR mixture composition 4

	Volume per reaction	Stock concentration	Final concentration
PrimeSTAR max	25 μL	-	-
RT-PCR-purified product	X μL (20 ng)	-	-
AdapterF	5 μL	10 μM/μL	1 μM/μL
IndexR	5 μL	10 μM/μL	1 μM/μL
Ultrapure water	15-X μL	-	-
  b. Perform PCR amplification using the program described in Table 14.

**Table 14:** PCR program 4

Steps	Temperature	Time	Cycles
Initial Denaturation	98°C	5 min	1
Denaturation	98°C	30 s	5–10 cycles
Annealing	52°C	30 s	-
Extension	72°C	1 min	-
Final extension	72°C	5 min	1
Hold	12°C	Forever
  c. Purify the PCR product using 1.0 × DNA beads, adding 20 μL beads per tube.
  d. Elute purified DNA in 20 μL Ultrapure water.
  e. Clean the NanoDrop pedestal with ddH_2_O, measure a baseline using 1 μL ddH_2_O, then determine DNA concentration using 1 μL purified product.
  f. Run gel electrophoresis using 4 μL purified product and 4 μL unpurified product.
  g. Expected result is a correct band at ∼330 bp and no primer-dimer (∼100 bp).

### Library sequencing and preliminary data processing

**Timing: 1 to 2 weeks (1 to 2 weeks for sequencing and 1 day for data processing)**

This step describes library sequencing and alignment of specific sequence patterns from raw FASTQ files. The files processed in the following steps consist of barcode sequencing data derived from the target regions. Read 1 contains barcode sequences originating from the rabies virus or spike-in control RNA, while Read 2 contains the region index and UMI sequences. The demonstration dataset was generated using NovaSeq S4 paired-end 150 bp sequencing, and the first 1 million reads were subsampled for analysis.

26. If the libraries are of the expected size, submit them for 150-bp paired-end sequencing on an Illumina NovaSeq S4 platform. For starter region barcode libraries, target ∼10 million reads per sample; for input region barcode libraries, target 30–40 million reads per sample.

***Note:*** Comparable next-generation sequencing platforms may also be used (e.g., the Illumina NovaSeq 6000).

27. Set parameters according to the library structure.

library(ShortRead) # version 1.64.0

library(Biostrings) # version 2.74.1

library(dplyr) # version 1.1.4

# If helper functions are in a separate file:

source("helpers.R")

# 1) Inputs and streaming parameters

fastq_r1 <-  "data/input_R1.fastq.gz"

fastq_r2 <-  "data/input_R2.fastq.gz"

block_size <- 1e5

stopifnot(file.exists(fastq_r1), file.exists(fastq_r2))

# 2) Define random patterns (must contain 'N') and mismatch allowances

read1_patterns <- list(bcv1 = "NNNACNNNGTNNNCGNNNTANNNCANNNTGNNN",  spk = "NNNGTNNNCANNNGCNNNATNNNGTNNNCANNN")

read1_mismatches <- list(bcv1 = 1, spk = 1)

read2_patterns <- list(

 region = "AAGCCGATCTGCNNNNNNNN",

 umi = "NNNNNNNNNNNNGATGACCCAGCC"

)

read2_mismatches <- list(region = 1, umi = 1)

28. Align sequencing reads using the specified patterns.

# 3) Stream FASTQ in blocks and accumulate parsed results

s1 <- ShortRead::FastqStreamer(fastq_r1, n = block_size)

s2 <- ShortRead::FastqStreamer(fastq_r2, n = block_size)

open(s1); open(s2)

tb_list <- list()

i <- 1L

repeat {

 r1 <- yield(s1); r2 <- yield(s2)

 # Stop when either stream is exhausted

 if (length(r1) == 0 || length(r2) == 0) break

 reads <- list(reads1 = r1, reads2 = r2)

 # 3.1) Align each pattern to reads (approximate matching supported)

 aln <- align_patterns_pair(reads, read1_patterns, read2_patterns, read1_mismatches, read2_mismatches)

 # 3.2) Extract matched segments and keep only the random bases (N positions)

 segs <- extract_aligned_segments(reads, aln)

 # 3.3) Convert extracted sequences/qualities into a flat table (one row per read-pair index)

 tb_i <- segments_to_table(segs, quality_suffix = "_q")

 # 3.4) Append chunk table (skip empty chunks to keep tb_list compact)

 if (!is.null(tb_i) && nrow(tb_i) > 0) {

 tb_list[[i]] <- tb_i

 i <- i + 1L

 }

}

close(s1); close(s2)

29. Split reads derived from barcoded rabies virus or spike-in controls based on the detected region and UMI sequences.

# 4) Split records for downstream counting

# Combine all chunks

tb <- dplyr::bind_rows(tb_list)

tb_bcv1 <- tb %>%

 filter(bcv1 != "", region != "", umi != "") %>%

 select(bcv1, bcv1_q, region, region_q, umi, umi_q)

tb_spk <- tb %>%

 filter(spk != "", region != "", umi != "") %>%

 select(spk, spk_q, region, region_q, umi, umi_q)

30. Remove reads containing low-quality randomer sequences and count the frequency of each unique record.

# 5) QC and counting

# QC rule: allow at most 0 bases below Q20 in each extracted field

df_bcv1 <- filter_by_quality(tb_bcv1, q_cutoff = 20, max_lowq = 0) %>%

 group_by(bcv1, region, umi) %>%

 summarise(dp = n(), .groups = "drop") %>%

 arrange(desc(dp))

df_spk <- filter_by_quality(tb_spk, q_cutoff = 20, max_lowq = 0) %>%

 group_by(spk, region, umi) %>%

 summarise(dp = n(), .groups = "drop") %>%

 arrange(desc(dp))

## Expected outcomes

This protocol was developed to generate and use barcoded rabies virus for tracing inputs to single neurons. The titer of virus produced using the one-step method is relatively lower than that obtained with amplification-based protocols. On average, one batch yields 60 μL of virus at a titer of 2 × 10^7^ IU/mL and contains more than 7,000 unique barcodes. The one-step method performs better for producing barcoded SAD-B19G strain, with approximately a 10-fold improvement in both titer and barcode diversity. However, in the mPFC, this strain yielded fewer input cells than the CVS-N2c strain, although this effect may be brain region dependent.

Per animal, we typically recovered approximately 60 starter cells with well-defined transcriptomic identities and unique barcodes, each receiving an average of 7.2 locally connected inputs. Among the 11 long-range brain regions examined, more than half of the starter cells received inputs from over four brain regions. However, these results may vary depending on the starter region analyzed. The resulting dataset enables investigation of the relationships between local connectivity, long-range inputs, and transcriptomic cell types.

## Limitations

A limitation of this protocol is the relatively low titer of CVS-N2c rabies virus and the still limited barcode diversity obtained from a single production batch. Multiple batches must be pooled to achieve sufficient barcode diversity for downstream tracing. In addition, a substantial fraction of cells is lost during single-cell suspension preparation, as well as during downstream library construction and data preprocessing. The recovery rate of starter cells is estimated to be only 5–10 percent. Another limitation is the lack of precise spatial information for the inputs, including both local single-cell inputs and bulk long-range inputs.

## Troubleshooting

### Problem 1 (related to step 1)

Excessive cell death is observed, the culture remains sparse until day 3, and the rabies virus titer is less than 1 × 10^7^ IU/mL.

### Potential solution

Prepare the maximum plasmid yield for rabies virus production using fresh reagents. Reduce the voltage or pulse duration in a stepwise gradient to determine the optimal transfection parameters.

### Problem 2 (related to step 1)

The rabies virus titer is less than 1 × 10^7^ IU/mL, despite a high post-transfection viability rate. Fluorescent clusters were rarely observed, even at 6 days post transfection.

### Potential solution

Increase the voltage or pulse duration in a stepwise gradient to determine the optimal transfection parameters.

### Problem 3 (related to step 2)

Input neurons are sparse in most of the input regions.

### Potential solution

Extend the expression period of the helper virus to 3–4 weeks. Split TVA and N2cG into separate vectors, such as *AAV-hSyn-DIO-N2cG* and *AAV-hSyn-DIO-TVA*. Perform a stepwise dilution of N2cG from 5 × 10^12^ to 5 × 10^11^ vg/mL.

### Problem 4 (related to step 3)

The number of recovered cells is fewer than 10,000 after single-cell suspension preparation, which is insufficient for single-cell sequencing, and cell clumps remain visible even after 20 passes through a 200-μm glass pipette tip.

### Potential solution

Extend the digestion duration and prepare the papain working solution using freshly activated papain.

### Problem 5 (related to step 3)

The number of recovered cells is fewer than 10,000 after single-cell suspension preparation, despite the absence of visible clumps after 10–15 passes through a 200-μm glass pipette tip.

### Potential solution

Increase the amount of dissected tissue; use low-protein-binding tubes and pipette tips to minimize cell loss.

### Problem 6 (related to step 4)

Helper virus transcripts are detected in only a few cells and at low read counts.

### Potential solution

Enrich helper virus transcripts from the cDNA, as described in step 19. Use nested primers targeting the helper virus cDNA insert, and perform a second round of PCR if necessary to specifically amplify low-abundance transcripts.

### Problem 7 (related to step 3)

More than 1,000 starter cells are observed by imaging in preliminary experiments, whereas fewer than 50 starter cells are recovered by sequencing.

### Potential solution

Verify co-expression of the helper virus and rabies virus and prioritize samples with robust co-expression for downstream single cell suspension preparation.

## Resource availability

### Lead contact

Requests for further information and reagents should be directed to and will be fulfilled by the lead contact, Hua-tai Xu (xuht@lglab.ac.cn).

### Technical contact

Technical questions on executing this protocol will be answered by the technical contact, Kang Tan (tankang@lglab.ac.cn).

### Materials availability

The cell lines and plasmids described in this paper are available from the lead contact upon request.

### Data and code availability

Original data have been deposited to Figshare: https://doi.org/10.6084/m9.figshare.31072744. The code used to analyze the data is also publicly available at the same Figshare repository.

## Acknowledgments

We thank Dr. Edward Callaway and Dr. Lei Jin for sharing their reagents for making rabies viruses and other members of the Xu lab for their discussions and technical support. This work was supported by grants from the National Science and Technology Innovation 2030 Major Program, grant no. 2021ZD0200100; the Lingang Laboratory, grant no. LG-GG-202201-01; and the 10.13039/501100003399Shanghai Municipal Science and Technology Major Project, grant no. 2018SHZDZX05.

## Author contributions

K.T. and H.-t.X. conceived the project. K.T. and Y.-q.W. prepared barcoded RV. K.T., Y.-q.W., and L.F. prepared samples. K.T. and H.-t.X. wrote the manuscript.

## Declaration of interests

The authors declare no competing interests.

## Declaration of generative AI and AI-assisted technologies in the writing process

During the preparation of this work, the authors used ChatGPT (OpenAI, versions 5.0 and later) in order to assist in editing text originally drafted by the authors, with the aim of improving clarity and readability. After using this tool, the authors reviewed and edited the content as needed and take full responsibility for the content of the published article.
